# Ignoring the Older Person’s Voice: Dignity, Ageism, and Epistemic Injustice in Geriatric Nursing Practice—A Nursing-Oriented Conceptual Review and Practice Framework

**DOI:** 10.3390/healthcare14162629

**Published:** 2026-08-19

**Authors:** Georgios Manomenidis, Charikleia Orfanidou, Christos Kleisiaris, Savvato Karavasileiadou, Panagiota Kazakou, Areti Nikiforou, Vasiliki Georgousopoulou

**Affiliations:** 1 Department of Nursing, Democritus University of Thrace, 68100 Alexandroupolis, Greece; xaraorf@gmail.com (C.O.); vgeorgousopoulou@yahoo.gr (V.G.); 2 Department of Nursing, University of Thessaly, 41500 Larissa, Greece; kleisiaris@uth.gr; 3 Department of Community and Psychiatric Mental Health Nursing, College of Nursing, Princess Nourah bint Abdulrahman University, Riyadh 11671, Saudi Arabia; 4 University General Hospital of Alexandroupolis, 68100 Alexandroupolis, Greece; giotakazakou77@yahoo.gr; 5 Blocks Rehabilitation Filoktitis, 19400 Athens, Greece; arenikiforou@gmail.com

**Keywords:** dignity, epistemic injustice, ageism, geriatric nursing, older adults, person-centred care, epistemic humility, shared decision-making, epistemic documentation

## Abstract

**Highlights:**

**What are the main findings?**

- Epistemic injustice helps explain how older adults may be formally respected yet still not believed, understood, documented, or allowed to influence decisions.
- The article proposes a five-principle framework for dignity-preserving geriatric nursing: credibility-oriented listening, narrative recognition, supported participation, relational decision-making, and epistemic documentation.

**What are the implications of the main findings?**

- Geriatric nursing education should address age-related credibility deficits, family substitution, and documentation practices that can silence older adults.
- Healthcare organizations and clinical governance can operationalize epistemic justice through assessment prompts, handover structures, supported communication pathways, and reflective review of cases labelled as refusal, confusion, or non-compliance.

**Abstract:**

**Background/Objectives:** Older adults may experience healthcare encounters in which their voices are discounted, redirected through family members, or interpreted through age-related assumptions about cognitive decline. Although such problems are often discussed through dignity, communication, ageism, person-centred care, or shared decision-making, this conceptual review argues that they also involve epistemic harms: harms that occur when older adults are not recognized as credible knowers of their own bodies, needs, values, histories, and care priorities. **Methods:** This article develops a conceptual and critical analysis of Fricker’s account of testimonial and hermeneutical injustice, later critiques of epistemic injustice, and the nursing and gerontological literature on dignity, person-centred nursing, documentation, dementia care, and shared decision-making. **Results:** The analysis distinguishes ordinary communication failure, institutional restriction of interpretive space, testimonial injustice, and hermeneutical injustice. It identifies interacting interpersonal and institutional mechanisms through which older adults’ knowledge may be discounted or rendered invisible and develops five theoretically derived principles: credibility-oriented listening, narrative recognition, supported participation, relational decision-making, and epistemic documentation. For each principle, the framework identifies the epistemic failure addressed, its added value beyond general person-centred practice, and responsibilities at both individual and organizational levels. **Conclusions:** An epistemic-justice lens does not replace dignity, person-centred care, shared decision-making, or professional judgement. It adds a focused analysis of credibility, interpretive authority, knowledge visibility, and whose account shapes care. The proposed framework is a conceptual and normative contribution that requires empirical evaluation before its effects can be established.

## 1. Introduction

It is widely recognized that the number of people aged 60 years and older is expected to double by 2050, while the population aged 80 years and above is expected to triple [1]. Accordingly, geriatric care is increasingly prioritizing preventive and personalized nursing for older adults with multimorbidity, functional decline, socioeconomic disadvantage, and complex psychosocial needs [2].

Importantly, dignity is widely regarded as a cornerstone of person-centred geriatric care and is commonly associated with respect, autonomy, privacy, self-determination, individuality, and meaningful participation in healthcare decisions [3]. Dignity-preserving care therefore involves more than meeting physical needs. It requires recognizing that older adults’ values, preferences, life histories, and interpretations remain relevant despite functional decline, limitations in activities of daily living, or loss of independence. Nevertheless, older adults may encounter situations in which they feel ignored, patronized, infantilized, or excluded from decisions affecting their lives [4]. For instance, an older patient’s report of pain may be dismissed as an inevitable consequence of ageing, and a resident’s preferences may be subordinated to institutional routines. A capable older adult’s concerns may also be discussed primarily with relatives rather than directly with the person. In these situations, harm is not only a failure of respect or autonomy; it is also a failure to recognize the older person as someone whose testimony, interpretation, and experiential knowledge matter, particularly when age-related stereotypes reduce the credibility assigned to their voice [5].

The philosophical concept of epistemic injustice offers a useful starting point for understanding this problem. Fricker [6] described epistemic injustice as a wrong done to individuals in their capacity as knowers. She distinguished testimonial injustice, in which prejudice reduces a speaker’s credibility, from hermeneutical injustice, in which inadequate interpretive resources hinder the understanding and communication of important experiences. In healthcare, epistemic injustice has been used to examine how patients may be discredited, misunderstood, silenced, or excluded from clinical decision-making processes [7,8]. This perspective is especially relevant to older adults because ageing is often associated with stereotypes of cognitive decline, dependency, reduced competence, resistance to change, or limited ability to engage with complex health information [9]. Such assumptions may be implicit rather than intentional, yet they can shape everyday clinical interactions. Healthcare professionals may unintentionally use simplified or patronizing language, direct questions to relatives, or interpret disagreement as confusion. They may also treat limited participation as evidence of incapacity rather than as a consequence of exclusion or inadequate support. In such cases, age-related stereotypes may operate as credibility deficits that weaken the authority given to older adults’ own accounts [10,11].

Recent studies have shown that these processes have direct implications for dignity. Particularly, older adults value being recognized, heard, included, and treated as unique persons rather than being reduced to diagnoses, dependency, or age categories [3,12]. When older adults are spoken about rather than spoken with, or when their interpretations are replaced by those of professionals or relatives, dignity may be undermined through epistemic exclusion [12,13]. Examining how older adults’ voices are ignored therefore has direct implications for nursing practice, particularly for comprehensive geriatric assessment. Nurses often spend more time with older adults than other healthcare professionals and play a central role in assessment, communication, advocacy, symptom management, documentation, and relational care. From this perspective, geriatric nursing is not only a practice of physical and emotional care but also an epistemic practice that recognizes, interprets, validates, and communicates patient knowledge within clinical decision-making [14,15].

Although dignity, person-centred care, shared decision-making, and relational autonomy provide established ethical and clinical accounts of respectful care, they do not always make the epistemic mechanism of exclusion explicit. An older adult may be addressed politely and invited to participate, yet still be assigned less credibility, have an experience translated into categories that alter its meaning, or find that professional or family accounts alone determine what enters the record and shapes the decision. Epistemic injustice therefore adds a focused analysis of whose testimony is believed, whose interpretive framework defines the problem, which forms of knowledge become institutionally visible, and how unequal epistemic authority can persist beneath formally person-centred language [6,7,8,16,17,18,19,20]. Geriatric nursing is a particularly relevant disciplinary lens because nurses repeatedly elicit, interpret, document, communicate, and act on older adults’ experiential knowledge across assessment, daily care, handover, care planning, and transitions [14,15]. This conceptual review therefore aims (1) to delimit testimonial and hermeneutical injustice from ordinary communication failure, clinical uncertainty, and justified professional constraints; (2) to examine interpersonal, relational, and institutional mechanisms through which older adults may be marginalized as knowers; and (3) to derive a nursing-oriented, five-principle framework for dignity-preserving care.

## 2. Materials and Methods

### 2.1. Review Design and Scope

This manuscript is a conceptual analysis and critical narrative review rather than an empirical study, systematic review, or scoping review. Its purpose is not to estimate prevalence, evaluate intervention effectiveness, or provide exhaustive evidence mapping, but to clarify the explanatory value and limits of epistemic injustice for geriatric nursing. The review brings three bodies of literature into conversation: (1) epistemic injustice and its critiques; (2) nursing and healthcare literature on person-centred care, dignity, relational autonomy, shared decision-making, and documentation; and (3) gerontological and dementia-care literature concerning ageism, dependency, communication, and participation. Literature identification and selection followed a purposive narrative approach, as described below. Given that the review was designed for conceptual clarification rather than exhaustive coverage or effect estimation, no PRISMA-based claim is made.

### 2.2. Information Sources and Literature Selection

To update and document the evidence base for this conceptual review, a supplementary narrative literature search was conducted in PubMed/MEDLINE, Scopus, and Web of Science on 1 May 2026 and covered records available through that date. Search terms were combined iteratively and included “epistemic injustice,” “testimonial injustice,” “hermeneutical injustice,” “epistemic oppression,” and “epistemic silencing,” together with terms relating to older people and geriatric care (“older adult*,” elder*, ageing, aging, geriatric*, and dementia), nursing and healthcare, and relevant care concepts, including dignity, person-centred care, patient voice, participation, shared decision-making, relational autonomy, narrative care, and documentation. Because the review was conceptual rather than systematic, the searches were refined as relevant theoretical concepts emerged. Database searching was supplemented by backward reference-list checking and forward citation tracking of key publications, together with targeted searches for foundational works on epistemic injustice and their subsequent applications in healthcare, nursing, and gerontology.

Sources were retained when they made a direct conceptual, theoretical, or empirical contribution to at least one of the review questions: how epistemic injustice affects older adults’ credibility and interpretive agency; how these processes relate to dignity, ageism, person-centred care, participation, relational autonomy, or documentation; and how the resulting insights could inform geriatric nursing practice. Foundational philosophical works and subsequent critiques were also included when they were necessary to develop or delimit the conceptual argument, even if they were not specific to geriatric care. Duplicate records and publications that did not substantively address ageing, healthcare, nursing, patient knowledge or participation, or provide a transferable conceptual contribution were excluded. Adjacent literature on ethics, decision-making capacity, long-term-care policy, and cultural or family involvement was retained only when it clarified an epistemic mechanism, a boundary condition, or a practice implication relevant to geriatric nursing. Sources addressing these fields without a substantive link to patient knowledge, credibility, interpretation, participation, or documentation were outside the review’s scope. Purposive selection inevitably involved interpretive judgement. G.M. conducted the initial literature identification and selection. To reduce reliance on one author’s perspective, co-authors from nursing and gerontological backgrounds reviewed the proposed source set, conceptual groupings, and their relevance to the framework. Differences concerning relevance or interpretation were resolved through discussion, and claims were repeatedly checked against foundational philosophical sources and healthcare applications. Formal independent duplicate screening was not undertaken; accordingly, this process supports conceptual credibility but not the reproducibility of a systematic review, and no claim of exhaustive or comprehensive coverage is made.

### 2.3. Conceptual Analysis and Framework Development

The conceptual synthesis was led by G.M. and V.G. through iterative comparative reading and a structured conceptual matrix. The matrix recorded for each retained source: (1) the form of epistemic problem described; (2) whether it operated primarily at an interpersonal, relational, or institutional level; (3) the knowledge at risk, including testimonial, embodied, narrative, value-based, or documented knowledge; (4) the relationship to dignity, person-centred care, participation, relational autonomy, or professional judgement; and (5) the nursing or organizational response proposed or implied.

Analysis proceeded in four stages. First, Fricker’s distinction between testimonial and hermeneutical injustice was used deductively as a sensitizing framework [6]. Second, later accounts of institutional epistemic justice, silencing, oppression, and willful hermeneutical ignorance were used to identify mechanisms not adequately captured by an interpersonal model [21,22,23,24,25]. Third, the authors compared these mechanisms with nursing and gerontological domains—dignity, person-centred care, experiential and narrative knowledge, supported participation, relational autonomy, and documentation—to generate candidate practice responses [3,16,17,18,19,20,26,27,28,29,30,31,32]. Fourth, candidate responses were compared, merged when they addressed the same mechanism, and retained as separate principles when they addressed a distinct epistemic risk and could be translated into observable individual and/or organizational practices.

This process produced five principles. Credibility-oriented listening was linked primarily to prejudicial credibility deficits; narrative recognition to loss or distortion of biographical and interpretive meaning; supported participation to remediable barriers that restrict a person’s contribution; relational decision-making to the fair and transparent weighting of patient, family, clinical, legal, and organizational knowledge; and epistemic documentation to the institutional visibility and continuity of the older adult’s account. S.K. and C.K. reviewed the matrix and proposed framework, after which all authors examined whether each principle was conceptually distinct, traceable to the literature, and consistent with the stated boundaries of epistemic injustice. Differences were resolved through discussion. Table 1 summarizes this derivation. The process was interpretive rather than a formal qualitative coding study, and the framework should therefore be understood as a theoretically derived proposal rather than an empirically validated model.

### 2.4. Analytic Boundaries

The analysis uses “epistemic injustice” in a bounded sense. Four situations should be distinguished. First, ordinary misunderstanding or inadequate communication may occur without identity prejudice or structural disadvantage; it still requires correction but is not, by itself, epistemic injustice. Second, an institutional failure of interpretive space occurs when time, forms, routines, or communication arrangements prevent fuller understanding. This becomes epistemically unjust when those arrangements systematically or repeatedly disadvantage older adults or other marginalized knowers and the disadvantage is treated as natural or unavoidable. Third, testimonial injustice occurs when an older adult’s credibility is reduced because of identity-linked prejudice—for example, when age, dementia, dependence, sensory impairment, or emotional expression is used as a shortcut for unreliability before individualized assessment [6,7,8]. Fourth, hermeneutical injustice, in the stronger Frickerian sense, requires more than difficulty describing an experience: it involves a structurally unequal gap in shared interpretive resources that places a marginalized person at a disadvantage in understanding or communicating a socially significant experience [6]. Drawing on post-Frickerian work, the analysis also recognizes institutional silencing, willful hermeneutical ignorance, and epistemic oppression when dominant practices repeatedly fail to engage with interpretive resources available from marginalized experience [21,22,23,24,25].

Emergencies, legal duties, safeguarding requirements, clinical uncertainty, resource limitations, and professional disagreement do not automatically constitute epistemic injustice, nor do they automatically justify excluding the older adult’s account. The relevant questions are whether the constraint is genuine and proportionate, whether reasonable communication support was attempted, whether age-linked assumptions influenced credibility, whether the person’s account was elicited and documented, and whether the rationale for overriding or limiting a preference was explained and reviewable [8,28,29,37]. Epistemic justice therefore does not require the older adult’s view always to prevail. It requires that the view is not downgraded in advance and that competing evidence is weighed transparently.

The framework is intended to complement, not replace, dignity, person-centred care, shared decision-making, relational autonomy, capacity assessment, or professional accountability. These approaches identify ethical goals and deliberative processes; epistemic justice adds diagnostic questions about credibility, interpretive authority, knowledge visibility, and whose account ultimately shapes the definition of the care problem [16,17,18,19,20].

## 3. Results

The conceptual findings are organized below in three linked parts: first, the relevance and limits of Fricker’s framework; second, the ways in which older adults’ experiential, embodied, narrative, and value-based knowledge may be marginalized in healthcare; and third, a nursing-oriented framework for epistemic justice in dignity-preserving geriatric care.

### 3.1. Fricker’s Framework: Continuing Relevance and Critical Extensions

Fricker’s account of epistemic injustice remains valuable for nursing philosophy because it identifies a form of harm that is not reducible to physical harm, lack of information, poor communication, or disrespectful behaviour, and because recent nursing scholarship has begun to examine how epistemic silence operates within nursing knowledge, education, and care relationships [38]. Its continuing relevance lies in showing that people may be wronged specifically in their capacity as knowers, either when their testimony is assigned reduced credibility or when their experiences cannot be adequately understood within available interpretive frameworks [6]. For geriatric nursing, this distinction remains useful because older adults may be harmed both when their pain, fear, preferences, or symptoms are not believed, and when experiences such as dependency, loneliness, loss of identity, or fear of institutionalization are not recognized as legitimate concerns within clinical conversations.

At the same time, Fricker’s framework should not be treated as complete. One important critique is that the original account focuses strongly on interpersonal credibility judgements and individual hearer virtues, while giving less attention to institutional, structural, and collective forms of epistemic exclusion. Anderson [21], for example, argues that epistemic justice should be understood not only as a virtue of individuals but also as a virtue of social institutions. This critique is particularly important for healthcare, where epistemic injustice is rarely produced only by one clinician’s attitude. It may also be embedded in documentation systems, time pressures, risk-management cultures, family-centred decision-making patterns, and institutional routines that determine whose accounts are recorded, circulated, silenced, or acted upon. Subsequent scholarship has also expanded Fricker’s original distinction between testimonial and hermeneutical injustice.

Dotson [22,23] draws attention to epistemic violence and epistemic oppression, showing how silencing may occur when speakers are not adequately heard or when communities are excluded from knowledge-producing practices. Pohlhaus [24] develops the concept of willful hermeneutical ignorance, emphasizing that dominant groups may fail or refuse to engage with interpretive resources developed from marginalized experience. Medina [25] further shifts attention toward epistemic resistance, shared responsibility, and the transformation of social conditions that make some voices difficult to hear. These developments are important for the present paper because older adults’ epistemic marginalization is not only a matter of being personally disbelieved; it may also arise from institutional forms of hearing, recording, interpreting, and deciding that make some experiences more visible than others.

This paper therefore uses Fricker’s concepts as a starting point rather than as a complete framework. Testimonial and hermeneutical injustice provide the basic conceptual structure for analyzing how older adults may be discredited or misunderstood in geriatric nursing. However, the argument is extended through later critiques that emphasize institutional conditions, relational knowing, silence, epistemic oppression, and the need for practical forms of epistemic repair. In this respect, the proposed framework of credibility-oriented listening, narrative recognition, supported participation, relational decision-making, and epistemic documentation is not simply an application of Fricker’s theory. It is a nursing-oriented development of epistemic justice adapted to the structural, relational, and institutional realities of geriatric care.

This extension is particularly important because recent healthcare scholarship warns against the uncritical transfer of epistemic injustice into clinical practice. Fundamental questions remain about how to distinguish epistemic injustice from diagnostic uncertainty, disagreement, risk assessment, professional responsibility, and unavoidable limits of healthcare systems [8]. The argument developed here therefore treats epistemic injustice as one explanatory lens among others. Its value lies in identifying a specific wrong, downgrading or constraining older adults as knowers while still recognizing that nurses must also act under clinical, legal, organizational, and evidential constraints.

A central limitation of a simple Frickerian application is that it may over-personalize epistemic injustice by focusing on whether an individual nurse believes or disbelieves an individual older person. In geriatric care, however, epistemic marginalization is often distributed across systems: assessment forms may privilege measurable risk over lived concerns; handovers may transmit diagnoses and tasks but not patient meanings; discharge pathways may compress complex preferences into institutional categories; and time-pressured routines may make narrative work appear optional. Anderson’s institutional account of epistemic justice, Dotson’s work on epistemic oppression and silencing, Pohlhaus’s account of willful hermeneutical ignorance, and Medina’s emphasis on epistemic resistance therefore help extend Fricker’s framework from individual virtue to institutional responsibility [21,22,23,24,25].

### 3.2. Older Adults as Epistemic Agents in Person-Centred Care

Older adults should not be understood only as recipients of care, passive beneficiaries of professional expertise, or individuals whose experiences require interpretation by others [26]. They are also epistemic agents: persons who possess knowledge about their own bodies, lives, values, relationships, and priorities. This knowledge should not be treated as secondary to clinical knowledge, because in geriatric care it is often essential for understanding symptoms, treatment preferences, quality of life, dependency, and dignity [27]. Older adults’ experiential knowledge is shaped across the life course through illness, ageing, family roles, work, loss, adaptation, social relationships, and previous encounters with healthcare systems. A life-course perspective is useful because current health, preferences, coping strategies, and meanings of care are influenced by accumulated experiences across earlier stages of life [39]. This helps explain why two older adults with similar diagnoses or functional limitations may understand care, risk, independence, or dignity in very different ways. For this reason, older adults’ own accounts should be treated as an important source of clinical and ethical understanding.

Bogaert’s critique of empowerment in person-centred healthcare is relevant here because it shows that programmes can be designed for patients while still lacking patient-developed concepts and patient-defined meanings [40]. In geriatric nursing, the same risk occurs when older adults are invited to participate only within categories already defined by professionals or institutions. Epistemic justice therefore requires not only asking older adults what they prefer but also being open to how they frame the problem, what they consider meaningful, and which forms of support they regard as dignity-preserving.

This claim is consistent with, but also extends, person-centred nursing. The Person-centred Nursing Framework highlights the importance of knowing the person, working with beliefs and values, authentic engagement, shared decision-making, and care environments that support power-sharing [16]. Epistemic justice specifies the knowledge-related condition for these ideals: the older person’s account must be treated not merely as a preference to be noted, but as a source of evidence and meaning that can shape what counts as the care problem and what counts as a reasonable care response.

Embodied knowledge is particularly important in later life because ageing is experienced through the body, including pain, fatigue, reduced mobility, sensory changes, vulnerability, and altered confidence in everyday activities [41]. Older adults’ accounts of discomfort, instability, fear of falling, exhaustion, or loss of bodily control may capture aspects of ageing that are not fully visible through clinical measurement and should therefore inform responsive and dignity-preserving care [42].

Narrative and value-based knowledge are also central to geriatric nursing. Older adults understand their current health and care needs in relation to their life stories, including previous illness experiences, family roles, losses, relationships, values, preferences, and expectations for the future. Narrative approaches help nurses understand the person behind the diagnosis and connect care decisions with the older adult’s biography, identity, and sense of meaning [33,34,35]. This is important because decisions about treatment, rehabilitation, institutional care, or end-of-life planning are often shaped by what older adults consider most important, such as independence, comfort, family presence, privacy, familiar routines, avoidance of suffering, remaining at home, and maintaining social roles. These priorities are not peripheral preferences; they can shape care goals, acceptable risk, treatment decisions, and perceptions of quality of life and dignity [43]. They may also differ from professional assumptions about safety, rehabilitation, or treatment benefit. Person-centred geriatric care therefore requires listening to how older adults define good care, acceptable risk, dignity, and quality of life within the context of their own lives [44].

Recognizing older adults as epistemic agents does not mean ignoring cognitive impairment or treating every expressed preference as proof of decision-making capacity. Communication ability, cognition, epistemic agency, and decision-making capacity are related but distinct. A person may have difficulty making a particular treatment decision yet still communicate reliable knowledge about discomfort, values, familiar routines, relationships, or acceptable quality of life; conversely, fluent speech alone does not establish capacity. Capacity assessment concerns the person’s ability in relation to a specific decision and should not be inferred solely from age or diagnosis [28,37]. Epistemic agency is broader: it concerns the person’s continuing ability to contribute knowledge and meaning to care. Nurses should therefore optimize hearing, vision, language, timing, environment, and communication support; seek the older adult’s account directly; and distinguish an assessed limitation from a remediable barrier before relying primarily on another person’s interpretation [28,29,37,45].

### 3.3. Embodied Ageing and Institutional Power

Ageing is not only a biological process but also a lived experience involving changes in physical capacity, mobility, sensory function, energy, appearance, and health status. Phenomenological accounts of illness emphasize that the body is not merely an object of medical observation but the medium through which individuals experience the world and construct meaning [42].

Dependency is particularly important in this context. The need for assistance with activities of daily living (ADLs), such as mobility, personal hygiene, eating, and toileting, as well as with medication management or communication, may be experienced not only as a practical limitation but also as a threat to dignity, control, and personhood [46]. Two older adults with similar levels of physical impairment may interpret dependency differently depending on their personal histories, relationships, cultural values, and expectations regarding ageing. For this reason, the older person’s own interpretation of dependency remains an essential source of knowledge for geriatric care.

Healthcare institutions can either support or weaken this epistemic position. Hospitals, rehabilitation units, and long-term care facilities are often organized around routines, risk management, documentation, and efficiency. Although these structures are necessary for safe care, they may also limit opportunities for older adults to express preferences and influence decisions [47]. Mealtimes, bathing schedules, medication routines, mobility restrictions, and visiting arrangements may be shaped more by organizational priorities than by individual preferences. In such settings, older adults may gradually lose not only practical control but also authority over how their own needs and experiences are understood.

Institutional epistemic harm can arise even when no individual professional intends to silence the person. For example, an electronic record may offer selectable labels such as “refused mobilization” but no prompt for the patient’s explanation. If an older adult declines morning mobilization because analgesia has not yet taken effect, successive handovers may reproduce “non-compliant” while the timing of pain and the person’s proposed alternative disappear. The resulting harm is not simply poor wording: the record converts one interpretation into institutional fact, influences later credibility judgements, and narrows future options [20,21,22,23,24,25,32]. Comparable mechanisms can occur when a handover contains diagnoses and risks but omits what matters to the person, when a family member’s account is copied forward without identifying its source, or when staffing and workflow make supported communication unavailable.

These mechanisms require responsibilities at more than one level. Individual nurses can ask, listen, verify sources, document the person’s words, and challenge prejudicial labels. Teams can structure handovers and case review so that patient goals, unresolved disagreement, and the source of each account remain visible. Organizations must examine form design, staffing and time allocation, access to interpreters and communication aids, capacity-assessment pathways, family-meeting procedures, and governance review of recurrent labels such as “refusal,” “confusion,” or “non-compliance.” A structurally produced problem should not be assigned to individual nurses alone.

Institutional power also shapes whose knowledge is treated as legitimate. Clinical assessments, diagnostic labels, cognitive scores, risk evaluations, and care plans are essential for safe and coordinated care, but they may overshadow the older person’s own account of pain, fear, discomfort, loneliness, or quality of life when professional categories are treated as more authoritative than lived experience [48]. An older adult’s concern may be treated as confusion, resistance, or difficulty adjusting, rather than as meaningful testimony about their experience of care. This risk is intensified by ageist assumptions that older people are less reliable, less capable, or less able to participate in complex decisions [1].

Embodied ageing and institutional power therefore create conditions in which older adults may be particularly vulnerable to epistemic injustice. Their knowledge may be displaced by professional assessments, family interpretations, institutional routines, or age-based assumptions. Accordingly, dignity-preserving geriatric care requires clinical knowledge to be integrated with the older person’s own experiential knowledge. Without this integration, older adults risk becoming objects of care rather than participants in the interpretation and direction of their own lives.

Recent syntheses of dignity in long-term care show that older adults’ dignity is shaped by interactions among institutional conditions, caregiving practices, and family involvement, and that understaffing, workload pressure, rigid routines, and the gap between person-centred care as a slogan and person-centred care as practice can threaten dignity [3]. These findings support the need to connect epistemic injustice with organizational conditions rather than treating failures of listening as purely interpersonal moral failures.

This analysis should not be read as a rejection of clinical expertise, risk assessment, or organizational coordination. Clinical measures, cognitive assessments, medication protocols, fall-prevention strategies, and discharge planning are necessary parts of safe nursing care. The epistemic problem arises when these professional or institutional forms of knowledge become so dominant that they erase the older adult’s own explanation of pain, fear, acceptable risk, dependence, loneliness, or quality of life. In such cases, institutional routines do not merely organize care; they also organize whose knowledge is heard and whose knowledge disappears.

### 3.4. From Epistemic Harms to Dignity Harms

An epistemic perspective suggests that dignity is threatened not only when older adults are treated disrespectfully, but also when they are denied recognition as credible contributors to knowledge about their own bodies, needs, values, and lives. Research on dignity in later life shows that older adults associate dignity with being recognized as persons, having their views taken seriously, participating in decisions, and maintaining agency despite illness or dependency [3,44]. From this perspective, epistemic harm can be understood as dignity and personhood harm: when an older person’s account is dismissed, replaced, or treated as less credible than professional or family interpretations, their status as an epistemic agent is diminished. This is particularly important when older adults are defined primarily through diagnoses, cognitive scores, risk assessments, or institutional labels, because their own interpretations of who they are and what matters to them may be pushed to the margins. In dementia care, this risk is intensified when cognitive impairment leads others to underestimate the person’s continuing ability to communicate preferences, emotions, discomfort, values, and meanings [49].

Importantly, dignity harms linked to epistemic injustice do not always arise from intentional disrespect. They may emerge through routine practices that appear efficient, protective, or benign. Professionals may simplify communication, relatives may answer on behalf of older adults, and institutional routines may prioritize documentation, risk management, or time pressures over dialogue. Over time, older adults may become less likely to voice concerns, express preferences, or challenge decisions if they learn that their perspectives have limited influence. Such silence should not automatically be interpreted as agreement or satisfaction; it may reflect repeated experiences of being unheard [7,50].

Viewing dignity through the lens of epistemic injustice therefore broadens conventional understandings of respectful care. Privacy, autonomy, and compassionate communication remain essential, but they are insufficient if older adults are not recognized as credible knowers of their own experiences. The challenge for geriatric nursing is therefore not simply to care for older adults, but to ensure that their voices remain visible, credible, and consequential within healthcare encounters.

### 3.5. Testimonial Injustice in Geriatric Nursing

Testimonial injustice occurs when a speaker is assigned reduced credibility because of prejudice related to their social identity [6]. In geriatric nursing, chronological age may function as such a prejudicial marker. Older adults may be perceived as forgetful, confused, dependent, resistant, or unable to understand complex health information before any individualized assessment of cognition, communication, or decision-making capacity has taken place. In this way, age-related stereotypes may create credibility deficits that weaken the authority of older adults’ own accounts of pain, symptoms, preferences, fears, and quality-of-life concerns [9].

In everyday practice, testimonial injustice may occur when an older person’s account is dismissed, minimized, or replaced by the interpretations of others. Pain may be treated as an inevitable consequence of ageing rather than as a symptom requiring assessment. Expressions of sadness, fear, fatigue, loneliness, or functional decline may be interpreted as “normal ageing” rather than as clinically and personally meaningful testimony. Family involvement may also become problematic when relatives are treated as the primary source of knowledge while the older person is bypassed despite being able to communicate.

This form of injustice is especially important in dementia care. People living with dementia may be assumed to lack meaningful insight or communicative capacity, even when evidence shows that many can continue to express preferences, emotions, discomfort, values, and choices, particularly when communication and decision-making are appropriately supported [28]. Cognitive impairment should therefore not be treated as epistemic absence. Nurses therefore need to distinguish between actual communication limitations and prejudicial assumptions about incapacity, using supportive communication strategies and careful capacity assessment rather than treating a dementia diagnosis as evidence of epistemic absence [37]. Addressing this form of injustice requires credibility-oriented listening: speaking directly to the older person, assessing capacity rather than assuming incapacity, documenting the patient’s own words, and treating expressions of pain, fear, sadness, refusal, or discomfort as meaningful forms of testimony.

### 3.6. Hermeneutical Injustice and the Experience of Ageing

Hermeneutical injustice concerns an unequal disadvantage in making sense of or communicating socially significant experience because shared interpretive resources are structurally inadequate [6]. It should not be used as a synonym for every misunderstanding, short consultation, or poorly explored concern. If a clinician simply fails to ask about loneliness or mishears a preference, the immediate problem may be inadequate communication. If a service provides no time or format for concerns outside biomedical categories, the problem may be an institutional restriction of interpretive space. The stronger claim of hermeneutical injustice is warranted when such arrangements place a socially marginalized group at a systematic disadvantage in understanding or communicating experience, or when dominant institutions repeatedly disregard interpretive resources developed from that group’s experience [6,21,22,23,24,25].

Experiences such as fear of dependency, existential loneliness, identity loss, or fear of institutionalization are therefore not inherently examples of hermeneutical injustice. They become relevant when prevailing clinical categories cannot adequately represent their meaning and this deficit predictably disadvantages older adults. For example, an older adult’s repeated statement that residential placement would mean “losing my life” may be recorded only as “anxious about discharge.” The first statement may refer to loss of home, social role, privacy, continuity, and identity, whereas the institutional label narrows it to an individual symptom and removes the practical meaning that should inform planning [20,36].

Narrative approaches, life-story work, person-centred assessment, and reflective listening can help repair this gap, but eliciting a story is not sufficient if the account is subsequently translated into categories that erase its meaning or has no influence on care [20,33,34,35]. Hermeneutical repair therefore requires both interpersonal interpretation and institutional arrangements that allow older adults’ own concepts, words, and priorities to enter assessment, documentation, handover, and deliberation.

### 3.7. Competing Epistemic Claims and Clinical Judgement in Geriatric Nursing

Clinical decisions rarely involve only one voice or one kind of knowledge. Nurses must weigh the older adult’s experiential testimony, family members’ biographical knowledge, clinical evidence, professional assessment, legal requirements, safeguarding duties, resource constraints, and organizational responsibilities. Epistemic justice does not make the older adult’s account automatically correct or decisive. Its minimum demand is that the account is not downgraded in advance because of age, dementia, dependence, sensory impairment, emotional distress, or slower communication, and that the reasons for the final judgement are transparent [5,8,28,29,37].

Communication ability should not be conflated with decision-making capacity, and impaired capacity for one decision does not erase the person’s knowledge of symptoms, comfort, values, identity, or preferred routines. When capacity is impaired or uncertain, the nurse should continue to seek and support the person’s contribution, identify the specific decision at issue, follow applicable legal and clinical procedures, and distinguish the person’s present account from family reports and professional conclusions [28,29,37]. The record should state who provided each account and how it influenced the decision.

Family members may occupy different roles: they may facilitate communication, contribute biographical knowledge, provide emotional support, hold legally recognized decision-making authority, or participate in substitute decision-making under applicable law. These roles should not be treated as interchangeable. A relative or advocate can strengthen epistemic agency by helping the older adult understand information or express a view, but can also displace that view if professionals address the supporter instead of the person or assume that family preferences represent the patient’s own. Nurses should therefore ask the older adult, whenever possible, how they want family involved; speak to the person first; identify the source of each claim; and monitor whether support is enabling or replacing participation [28,29,30].

Cultural variation requires particular care. In family-centred contexts, autonomy may be understood relationally and an older adult may actively prefer collective deliberation or deferential family involvement. Epistemic justice should not impose an individualistic model by treating chosen interdependence as loss of agency. Equally, “culture” should not be used to presume that a particular older adult wants relatives to decide or to obscure disagreement within a family. The relevant standard is culturally responsive inquiry: ask the person how decisions are usually made, who should be involved, and whether the current arrangement reflects their values and wishes [30,51].

Safety, regulation, or workflow may justify limiting a preference, but naming such a constraint is not sufficient. Consider an older adult who wishes to walk independently despite a high fall risk. A credibility-preserving response is not to accept the request uncritically, but to assess capacity and risk, explore what independent walking means to the person, consider supported alternatives, explain any proportionate restriction, document both the person’s view and the clinical rationale, and set a review point. Epistemic harm is more likely when “safety” closes discussion, age or diagnosis substitutes for individualized assessment, feasible support is not considered, or the older adult’s explanation disappears from the record.

Epistemic humility supports this adjudicative work by requiring awareness of the limits of professional and family knowledge and active inquiry into missing perspectives [5]. It does not require abandonment of professional responsibility. It requires that disagreement be handled through supported communication, source clarification, proportionality, transparent reasoning, documentation, and review rather than through premature closure.

### 3.8. Epistemic Justice as a Framework for Dignity-Preserving Geriatric Nursing

The preceding analysis supports a five-principle nursing framework that links distinct epistemic mechanisms to individual and institutional responses. The framework is conceptual and normative: it proposes practices that may support dignity but does not claim that their effects have already been established. Table 1 shows how the principles were derived; Table 2 specifies the epistemic failure addressed, the incremental contribution of the epistemic-justice lens, and responsibilities at different levels.

Dignity and person-centred care identify ethical and clinical goals, including recognition of personhood, biography, rights, values, partnership, and participation [16,17,18,19]. Epistemic justice adds a diagnostic lens: it asks whether identity prejudice alters credibility, whether available categories distort marginalized experience, whether participation barriers are remediable, whose knowledge receives interpretive authority, and which accounts become durable and actionable through organizational systems [6,7,8,20,21,22,23,24,25]. Thus, a service may use person-centred language yet remain epistemically unjust if it politely records a preference but assigns it no credibility, translates it in a way that changes its meaning, or excludes it from the reasoning that determines care. The principles should be applied together rather than as a checklist. Listening without supported communication may reproduce exclusion; narrative elicitation without faithful documentation may have no institutional effect; and documentation without transparent deliberation may preserve a voice without allowing it to influence care.

#### 3.8.1. Credibility-Oriented Listening

The first principle requires nurses to approach older adults’ accounts as initially credible and evaluate them on their content and context, without allowing age, diagnosis, disability, or dependence to predetermine their credibility. Any adjustment in credibility should rest on an individualized, decision-relevant assessment—such as an identified communication barrier, delirium, or a documented inconsistency—and should prompt clarification or support rather than wholesale dismissal. Age-related stereotypes may lead healthcare professionals to question an older person’s testimony before assessing actual communicative or cognitive ability [11]. Credibility-oriented listening challenges this tendency by treating descriptions of pain, discomfort, fear, sadness, preferences, refusal, or priorities as clinically and ethically meaningful. In practice, this involves speaking directly to the older person before relying on family members, checking whether sensory barriers such as hearing or vision difficulties affect communication, allowing sufficient response time, avoiding elderspeak or infantilising language, and distinguishing cognitive impairment from unsupported communication. It also requires awareness of credibility excesses given to professionals or relatives, because older adults may be displaced not only when they are believed too little, but also when others are believed too quickly. Examples other than pain include treating new fatigue as ‘normal ageing,’ interpreting a request to delay bathing as confusion, or dismissing concern about medication effects because the person is described as anxious. In each case, the epistemic question is whether the account was assessed on its content or discounted through an age-coded assumption.

#### 3.8.2. Narrative Recognition

The second principle is narrative recognition. Older adults should not be understood only through symptoms, diagnoses, or care needs, but as persons whose identities are shaped by life histories, relationships, values, achievements, losses, and expectations for the future. Narrative recognition requires nurses to interpret the older person’s situation within the context of biography as well as biomedical information [31]. This matters because treatment preferences, fears of dependency, attitudes toward institutional care, and understandings of dignity often emerge from lived experience rather than from clinical indicators alone. At the same time, narrative recognition should not be treated as a pleasant add-on or as a simple technique for collecting life stories. Naldemirci et al. [20] show that narrative elicitation in person-centred care can make credibility deficits visible and give voice to silenced groups, but it does not by itself remove structural and positional inequalities in clinical communication. Nurses therefore need reflective awareness of which narratives are readily heard, which are dismissed as unrealistic or excessive, and which are translated into institutional categories in ways that may weaken the patient’s original meaning.

#### 3.8.3. Supported Participation

The third principle is supported participation. Epistemic justice requires that older adults remain involved in decisions about their care to the greatest extent possible, even when sensory impairment, mild cognitive decline, fatigue, or communication difficulties are present [29]. Participation should not be treated as an all-or-nothing capacity. It can often be supported through appropriate communication strategies and environmental adjustments. Supported participation may include allowing additional time for responses, using written or visual information, reducing background noise, ensuring hearing aids or glasses are available, checking understanding, repeating information when needed, and revisiting conversations over time. The presence of cognitive or physical limitations should not automatically justify exclusion. Instead, nurses have a responsibility to create conditions that make participation possible. ‘Sufficient support’ means support proportionate to the observed barrier—for example, ensuring hearing aids and glasses are available, using an interpreter rather than a relative when language is the barrier, moving a complex discussion away from a noisy environment, revisiting a decision after analgesia or recovery from delirium, and checking understanding using accessible language. It does not require indefinite delay when urgent action is necessary.

#### 3.8.4. Relational Decision-Making

Relational decision-making distinguishes the older adult’s contribution from the different supportive, informational, legal, and substitute roles that families or advocates may hold. Its application to capacity, cultural variation, safety, and competing claims is detailed above. The central requirement is not that one voice always dominate, but that sources and roles are identified, support is attempted, and reasons for weighting competing claims are transparent and documented [28,29,30,37].

#### 3.8.5. Epistemic Documentation

The fifth principle is epistemic documentation. Healthcare documentation is not a neutral administrative task. What is documented becomes visible, legitimate, and actionable within healthcare systems. What is not documented may be forgotten, ignored, or excluded from subsequent decisions. Epistemic documentation requires nurses to record not only clinical observations but also the older person’s expressed wishes, concerns, fears, preferences, goals, and interpretations of their own situation [32]. When possible, documentation should preserve the patient’s own words. This allows the older person’s voice to remain present across shifts, multidisciplinary discussions, care transitions, and institutional routines. Epistemic documentation is especially important because documentation converts some forms of knowledge into institutional memory while leaving others vulnerable to disappearance. Person-centred documentation studies show that records can either preserve or flatten patient narratives depending on whether goals, values, preferences, and contextual meanings are documented alongside clinical facts [20,32]. Documenting the patient’s own words is therefore not merely administrative; it is a dignity-preserving epistemic practice. For example, “declined shower; stated that morning care causes severe fatigue and requested evening assistance” preserves testimony and an actionable alternative, whereas “uncooperative with hygiene” converts an interpretation into a stigmatizing institutional label. Documentation should also identify whether a statement came from the patient, a relative, direct observation, or professional inference [20,32].

Taken together, these five principles provide a practical framework for integrating epistemic justice into dignity-preserving geriatric nursing. This framework positions geriatric nursing as both a caring and epistemic practice, highlighting how nurses influence whose voices are heard, whose experiences are recognized, and whose knowledge shapes clinical decisions.

## 4. Discussion

The framework’s principal contribution is to identify a knowledge-related mechanism through which dignity and person-centred care may fail. Respectful language, an invitation to participate, or a completed preference field does not ensure epistemic justice if age-linked assumptions reduce credibility, institutional categories distort meaning, or the older adult’s account has no influence on assessment, documentation, or decisions. This lens therefore complements rather than rebrands established approaches: person-centred care specifies the desired relationship and process, whereas epistemic analysis tests how credibility, interpretive authority, and knowledge visibility are distributed within that process [16,17,18,19,20].

Related work has applied epistemic-injustice theory to paediatric nursing, particularly in relation to children’s rights, developmental forms of expression, and the risk that children’s voices are displaced by adult interpretation [52]. The present analysis extends this line of nursing scholarship to the distinct context of later life, where ageism, dependency, cognitive and communication changes, family involvement, and institutional processes may create different patterns of epistemic vulnerability. Its specific contribution is to translate these geriatric concerns into a framework that addresses both interpersonal and organizational mechanisms of epistemic exclusion.

The analysis also shifts responsibility beyond individual communication. Nurses can speak directly to the older adult, provide proportionate communication support, verify the source of claims, document the person’s words, and make disagreements visible. However, these actions depend on institutional conditions. Assessment forms, electronic records, handover templates, staffing, interpreter and communication-aid access, family-meeting procedures, capacity pathways, and governance review determine whether experiential knowledge can enter and remain within the care system [20,21,22,23,24,25,32,47]. Implementation should therefore pair education with organizational redesign; otherwise, structural epistemic problems may be recognized while responsibility for repair is placed mainly on individual nurses.

For education and practice development, scenarios should extend beyond repeated examples of pain dismissal. Cases may involve fatigue being normalized as ageing, a request to postpone care being labelled confusion, fear of residential placement being reduced to anxiety, a family member answering despite the person’s ability to respond, or a fall-prevention restriction imposed without exploring acceptable risk. Debriefing can ask: What knowledge was offered? Was credibility affected by identity-linked assumptions? What support was attempted? Who interpreted the problem? Which account entered the record? How were safety, legal duties, and competing evidence weighed? These questions connect the abstract concepts to observable reasoning and practice.

Organizational implementation could include prompts for the patient’s own words and “what matters most”; explicit source attribution in records; handovers that include goals and unresolved disagreement; access to interpreters and communication aids; review points after delirium, pain, or fatigue has improved; and case review of recurrent labels such as “refusal,” “confusion,” “unrealistic preference,” or “non-compliance.” These strategies are proposed applications, not proven interventions. Their effects on patient participation, documentation quality, decision concordance, dignity, and staff practice require empirical evaluation.

### Limitations and Future Research

This article is a conceptual and critical narrative review, not a systematic or scoping review. Source selection was purposive, no formal risk-of-bias assessment was undertaken, and the interpretive synthesis may reflect the authors’ disciplinary perspectives despite co-author review and repeated comparison with foundational and applied literature. The five principles are theoretically derived and have not been validated as a measurement model, intervention, or standard of best practice; the effects stated in Table 2 are therefore proposed rather than established. The framework also requires contextual adaptation. Epistemic risks may differ across acute care, rehabilitation, community care, long-term care, palliative care, and dementia services. Cultural expectations concerning ageing, autonomy, dependency, and family involvement may support chosen interdependence in some settings while enabling substitution of the older adult’s voice in others. The analysis offers culturally responsive questions but does not provide a comprehensive cross-cultural theory. Legal definitions and procedures concerning capacity and substitute decision-making also vary across jurisdictions and should be applied according to local law and policy. Future qualitative and observational studies should examine how older adults, including people with cognitive or communication impairment, experience credibility, interpretation, family involvement, documentation, and disagreement in real encounters. The framework’s content validity, feasibility, and acceptability should first be evaluated with older adults, family caregivers, nurses, and interdisciplinary professionals. Subsequent studies could test educational and organizational applications using outcomes such as supported participation, source-attributed documentation, concordance between stated goals and care plans, review of restrictive decisions, and patient-reported dignity.

## 5. Conclusions

Epistemic injustice offers a bounded explanation of how dignity may be undermined when older adults are formally respected yet assigned less credibility, interpreted through categories that erase meaning, or excluded from the knowledge that shapes care. It should not be used to label every misunderstanding, disagreement, or justified clinical restriction. Its added value lies in examining identity-linked credibility, unequal interpretive resources, source and authority, and the institutional visibility of patient knowledge. The proposed five-principle framework links these mechanisms to credibility-oriented listening, narrative recognition, supported participation, relational decision-making, and epistemic documentation at both individual and organizational levels. It does not require the older adult’s account always to prevail; it requires that the account is elicited with reasonable support, not downgraded in advance, and weighed transparently alongside clinical, family, legal, and safety considerations. The framework remains conceptual and should be empirically evaluated before being treated as established practice.

## Figures and Tables

**Table 1:** Conceptual derivation of the five-principle framework.

Conceptual Source/Domain	Epistemic Mechanism Identified	Practice Question Generated	Resulting Principle
Testimonial injustice and ageism [1,6,9,10,11]	Prejudice-based credibility deficit assigned before individualized assessment	Is the older adult’s account being assessed on its content, or downgraded because of age, diagnosis, disability, or dependence?	Credibility-oriented listening
Hermeneutical injustice, narrative care, and experiential knowledge [6,20,22,23,24,25,27,33,34,35,36]	Marginalized experience is difficult to express or is translated into dominant categories that alter or erase its meaning	Does the care process provide shared language and interpretive space for the person’s biography, values, losses, fears, and priorities?	Narrative recognition
Dementia care, supported decision-making, and shared decision-making [28,29,37]	Remediable sensory, cognitive, environmental, or procedural barriers restrict contribution and are mistaken for absence of agency	What support could enable the person to communicate knowledge or participate before another voice substitutes for them?	Supported participation
Relational autonomy and competing epistemic claims [5,28,29,30]	Patient, family, clinical, legal, and organizational accounts are weighted opaquely or one source is privileged in advance	Have the different roles and forms of evidence been distinguished and weighed transparently without treating the patient’s account as either automatically decisive or automatically inferior?	Relational decision-making
Institutional epistemic justice and documentation [14,15,20,21,22,23,24,25,32]	Records, handovers, workflows, and governance determine which knowledge becomes visible, durable, and actionable	Does the older adult’s account survive documentation, handover, team discussion, and care transitions, and can exclusions be reviewed?	Epistemic documentation

**Table 2:** Proposed epistemic-justice framework for dignity-preserving geriatric nursing.

Principle	Specific Epistemic Failure	What the Epistemic Lens Adds	Individual/Team Practice	Organizational Responsibility and Proposed Effect
Credibility-oriented listening	Age, diagnosis, disability, or dependence-linked credibility deficit before individualized assessment	Makes prejudicial weighting of testimony visible, even when communication appears respectful	Address the older adult directly; check hearing, vision, language, fatigue, delirium, and communication support; assess the content of the account and identify its source	Provide communication resources and audit recurrent dismissal or age-coded labels. Proposed effect: the person’s testimony is more likely to receive fair initial consideration
Narrative recognition	Biography, values, and lived meanings are flattened or translated into dominant clinical categories	Examines whose interpretation defines the problem, not merely whether preferences were collected	Ask what the experience means, what matters most, and how history, roles, losses, and relationships shape the concern; preserve key wording	Include narrative fields and review whether patient meanings influence plans. Proposed effect: experiential meaning is more likely to remain visible and consequential
Supported participation	Remediable sensory, cognitive, environmental, or procedural barriers are mistaken for absence of agency	Distinguishes lack of support from lack of meaningful contribution or decision-making capacity	Allow time; reduce noise; provide glasses, hearing aids, interpreters, accessible information, and repeated conversations; revisit after reversible impairment improves	Ensure access to communication aids, interpreters, capacity pathways, staffing, and suitable environments. Proposed effect: participation is more likely to reflect actual ability rather than unsupported conditions
Relational decision-making	Patient, family, clinical, legal, and organizational claims are conflated or weighted opaquely	Clarifies source, role, credibility, and authority; prevents both automatic patient primacy and automatic patient downgrading	Ask the person how family should be involved; distinguish supporters, informants, authorized representatives, and substitute decision-makers; explain conflicts and rationale	Standardize family-meeting, escalation, ethics-consultation, and review procedures. Proposed effect: competing claims are more likely to be weighed transparently and proportionately
Epistemic documentation	The older adult’s account disappears or is converted into an unqualified institutional label across records and transitions	Shows how records create institutional memory and determine which knowledge becomes actionable	Attribute sources; record the person’s own words, goals, concerns, disagreement, support used, and rationale for decisions	Design records and handovers to preserve patient voice, source attribution, unresolved disagreement, and review points; audit labels such as “refusal” or “non-compliance.” Proposed effect: the person’s knowledge is more likely to survive shifts, teams, and transitions

## Data Availability

No new data were created or analyzed in this study. Data sharing is not applicable to this article.
